# Restorative Effects from Green Exposure: A Systematic Review and Meta-Analysis of Randomized Control Trials

**DOI:** 10.3390/ijerph192114506

**Published:** 2022-11-04

**Authors:** Song Song, Ruoxiang Tu, Yao Lu, Shi Yin, Hankun Lin, Yiqiang Xiao

**Affiliations:** 1 State Key Laboratory of Subtropical Building Science, South China University of Technology, Guangzhou 510641, China; 2 School of Architecture, South China University of Technology, Guangzhou 510641, China; 3 School of Architecture and Urban Planning, Guangdong University of Technology, Guangzhou 510006, China

**Keywords:** green space, mental health, restorative benefit, meta-analysis, randomized controlled trials

## Abstract

Despite growing research on green space and health benefits, the body of evidence remains heterogeneous and unclear. A systematic review and meta-analysis of studies of randomized controlled trials (RCTs) with high evidence levels are deemed timely. We searched Scopus, PubMed, Embase, and Web of Science for the literature up to January 2022 and assessed bias using the Cochrane Risk of Bias tool 2.0. We calculated joint impact estimates for each green space exposure assessment technique using random and fixed effects models. Compared to non-green space situations, green space exposure was related to decreased negative feelings, such as fatigue −0.84 (95% CI: −1.15 to −0.54), and increased levels of pleasant emotions, such as vitality 0.85 (95% CI: 0.52 to 1.18). It also lowered physiological indicators, including heart rate levels, by 0.60 (95% CI: −0.90 to −0.31). Effect sizes were large and statistically significant, and the overall quality of the evidence was good. Existing RCTs on greenspace exposure pay insufficient attention to older and adolescent populations, different ethnic groups, different regions, and doses of greenspace exposure interventions. More research is needed to understand how and how much green space investment has the most restorative benefits and guide urban green space planning and renewal.

## 1. Introduction

Urbanization and climate change have placed a growing strain on current urban green space infrastructure, as well as increased morbidity from mental illnesses [1]. Moreover, public health crises, high-density urban growth dynamics, lifestyles reliant on motor vehicles, and a lack of outdoor activities diminish urban inhabitants’ access to the natural environment, increasing public mental health issues and even causing chronic illnesses [2,3]. One in five people in the United Kingdom reported depression during the COVID-19 pandemic in the first quarter of 2021, more than twice as many as before the pandemic [4]. As such, there is an urgent need for space and places for physical and mental health rehabilitation as the severity of these health illnesses that afflict contemporary metropolitan populations increases. Policymakers, designers, planners, and other practitioners confront the difficulty of planning green space and maintaining and enhancing natural resources that are vital for sustaining and promoting human well-being.

In recent years, evidence regarding urban green space promoting mental health has accumulated. The earliest evidence can be connected to Kaplan’s Restorative Environment and Attention Restoration Theory (ART) [5,6,7]. Accordingly, vegetation and other natural elements might stimulate involuntary attention, thereby restoring autonomous attention and the neurocognitive mechanisms on which it depends. During the same time frame, Ulrich’s Stress Recovery Theory (SRT) proposed that exposure to or observation of vegetation and other natural elements might induce pleasant emotions and temporarily reduce stress levels in highly stressed individuals and that stress or stress relief is a major mechanism for the restorative effects of natural landscapes [8,9]. There are two major types of current research on the association between green space and mental health. First, the psychological health impacts of various forms of green space, with the majority of early research concentrating on the therapeutic effects of natural green space such as forests, such as shinrin-yoku (forest bathing) [10]. Recent research has focused on community green space, parks, streets, and green campus areas [11,12]. Most studies indicate that the greater the proportion of natural elements in a green environment, the greater the mental health benefits [13]. Second, research on the mental health impacts of diverse groups in green space has concentrated chiefly on adolescents and middle-aged adults, with a recent shift toward older persons [14,15]. In general, several studies demonstrate the great health potential of green surroundings or green areas. Most of these studies have raised awareness of the health-protective advantages of proximity to green space [16].

Reports supporting the favorable health impacts of green space are fast accumulating, and there is an urgent need for a timely quantitative synthesis of the current research. Meta-analysis is one of the most often utilized research methodologies to assess interventions and therapies. It can measure the magnitude of the health impacts of green space initiatives by combining the evidence from several studies. Many earlier systematic review studies included only narrative summaries of the results of many studies [17], lowering their usefulness for the determination of which green space exposure strategies are likely to be the most successful. Several recent meta-analyses have pooled studies from multiple research designs with effect sizes that did not focus on randomized controlled trials (RCT) [18,19], limiting the validity of the reviews’ findings and making it challenging to determine the extent to which green space is beneficial for mental health. A recent meta-analysis of RCTs analyzed the effects of green and blue exercise on quality of life and included eight RCTs [20]. However, this review included studies from diverse populations (e.g., chronic obstructive pulmonary disease, chronic post-traumatic stress disorder, fibromyalgia, schizophrenic patients, and older adults), making it difficult to determine the utility of green space interventions in the general adult population. In addition, existing reviews have not addressed issues pertaining to the dose of direct green space exposure associated with health improvement, diminishing the credibility of claims that there is sufficient evidence to support the implementation of green space exposure-based interventions on a large scale.

A lack of research on the degree of green space’s mental health effects impedes the development of green intervention solutions. Quantitative aggregation of relevant research may not only greatly contribute to the study of green space and health but also highlight the potential of green space to benefit health. Health and well-being are vital in city planning [21,22]. A quantitative synthesis of the findings may guide suggestions to help the future integration of health into urban planning, as well as green space management and public health policy. Based on the above, the purpose of this research was to determine the intensity of the effects of direct green space exposure on restorative indices of physical and mental health. We conducted a meta-analysis of current RCTs of green space real-space exposure treatments in the general population to determine the physiological and psychological impacts of green space real-space experiments. To increase the homogeneity of the interventions, “green space” included only public green spaces in this study, excluding private gardens, indoor nature, and virtual nature. This study addresses the following research questions: (1) What effect do direct green space exposure activities have on the general population’s mental health? (2) What kinds of sensitive indicators indicate the physiological and psychological effects of direct exposure to green space? The results of this meta-analysis and review might be used to support initiatives and policies targeted at urban green space renewal and management as part of a wider effort to improve public health.

## 2. Materials and Methods

The review was conducted in accordance with the Preferred Reporting Items for Systematic Reviews and Meta-Analyses (PRISMA) statement [23]. We developed a systematic review protocol prior to the start of the review, which was registered with PROSPERO (CRD42022339558) on 29 June 2022, and is available at https://www.crd.york.ac.uk/prospero/display_record.php?RecordID=339558. All of the review team members followed the protocol established at the beginning of the review.

### 2.1. Search Strategy

On 6 December 2021, four electronic databases were searched for literature: Scopus, PubMed, Embase, and Web of Science. The search terms were (green or blue or nature* or outdoor or park or open space) and (physical activity* or exercise* or walking* or cycling*), referencing two earlier review articles [18,20]. The “blue space” was included in the search terms to cover natural environments that combine blue and green, such as grasslands by a pond in a park. Details on the terms and search procedure are provided in the Appendix A. In each of the four databases, only articles reporting RCTs were allowed. To increase the number of potential publications, the publication year was not a restriction. In addition, we examined the cited and referenced literature of the relevant studies to complement the research, using a final search date of 22 January 2022.

### 2.2. Study Selection

EndNote X9 software (Philadelphia, PA, USA: Clarivate) was applied to remove duplicate articles from the four databases. All of the authors screened, evaluated, and selected full-text articles for the relevance of the topic and based on the inclusion and exclusion criteria.

Inclusion criteria were as follows: (1) the research design was RCT; (2) subjects were from the general population; (3) the intervention of the experimental group involved green space exposure; (4) the outcome was one regarding the restorative benefits (physical or mental); and (5) the results contained sufficient quantitative data for further statistical analysis.

Exclusion criteria were as follows: (1) the research design was not RCT, including observational studies, review or narrative articles, only study protocols, and qualitative studies; (2) participants were from special populations, such as atopic patients, pregnant women, or special age groups; (3) the intervention did not involve exposure to a real green environment or was only a virtual environmental experience; (4) the outcome variable was not related to health restorative benefits; and (5) there was no clear quantitative data or incomplete data for the outcome variable.

### 2.3. Data Extraction

According to the requirements of the research, the data extraction and input processes were each carried out by two researchers in a way that was both independent and double-blind. The data that were extracted included information on the first author, the year of publication, the sample size of both the experimental and control groups, the age and gender of the participants, the intervention protocol (including time, frequency, and periodicity), and outcome indicators for both the experimental and control groups. If there were any conflicts over the article’s data extraction and input processes, a third researcher was brought in to examine and discuss the situation until an agreement was reached.

### 2.4. Risk of Bias Assessment

Two authors individually assessed the quality of the included RCT studies using the Cochrane Risk of Bias Assessment Tool (RoB) 2.0. In each trial, five categories were evaluated: randomization process, deviations from the intended interventions, missing outcome data, measurement of the outcome, and selection of the reported result. All of the domains were classified as low risk, some concern, or high risk.

### 2.5. Statistical Analysis

In this study, a meta-analysis was performed using Stata14 software. The studies were first tested for heterogeneity. I^2^ values were used to assess heterogeneity between studies, with 25% reflecting low heterogeneity, 50% moderate heterogeneity, and 75% high heterogeneity. If heterogeneity was greater than 50%, then a random effects model with Hedge’s g coefficient and a 95% confidence interval was chosen to pool effect sizes, and finally, forest plots were used to visualize the results. Cohen (1988) guided the interpretation of the effect sizes of 0.20, 0.50, and 0.80, which indicate small, medium, and large effects, respectively [24]. Significance tests were then performed to determine the significance of the effects, with a *p* value < 0.05 being considered statistically significant. Next, sensitivity analysis was performed to examine the stability of the studies. Finally, the Egger regression test was used to statistically evaluate publication bias.

## 3. Results

### 3.1. Study Selection

**Figure 1:**
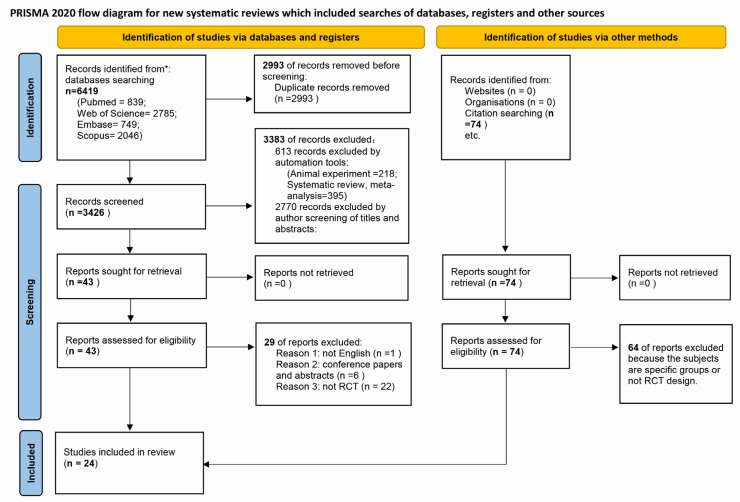
PRISMA 2020 flow diagram for the systematic reviews on the restorative benefits of exposure to green space. * According to the updated guideline from the PRISMA 2020 statement. Note: From [25].

A total of 6419 articles were identified by searching through four databases. After duplicate removal, 3426 items were retained and entered into the title and abstract screening step. At the end of the title and abstract screening step, we obtained 43 articles. Next, 43 full-text articles were independently evaluated for eligibility. According to the exclusion criteria, 29 articles were excluded for several reasons, including a non-English-language study, six conference papers or abstracts, and 22 non-RCT trial designs; 14 articles were retained. We also found 74 other articles after searching the references and citations of the selected studies, from which we retained 10 articles. Finally, we included 24 studies (14 from databases searching and 10 from references and citations search) in the review (Figure 1).

### 3.2. Study Characteristics

A total of 24 studies were considered for inclusion in this research, and Table 1 provides an overview of the fundamental aspects of the studies that were considered. The data of 1844 individuals were included in the statistical analysis. Their age range was 19–48 years, with 17 of the 24 studies having a mean participant age clustered in the 19–25 years range. Moreover, half of the studies were of mixed gender, with five being all-female, four all-male, and one not specifying the gender of the participants. The 24 RCTs consisted of 10 parallel studies and 14 crossover studies. The interventions consisted of exposing participants to real green space as well as activities. There were slight differences in intervention procedures across studies. First, the majority of the trials used a one-time, short-term intervention. Eleven trials were 15–20 min long, 10 were 30–60 min, and one was 5 min. The remaining two trials were long-term, with a total intervention duration of 8 weeks. Second, the intervention activities were predominantly walking and viewing, with cycling in two trials and a combined fitness program in one trial. Third, regarding the type of green space, 15 of the 24 studies were conducted in forests, while the rest were conducted in urban parks, grasslands, woodlands, country lanes, and green façade.

### 3.3. Risk of Bias

**Figure 2:**
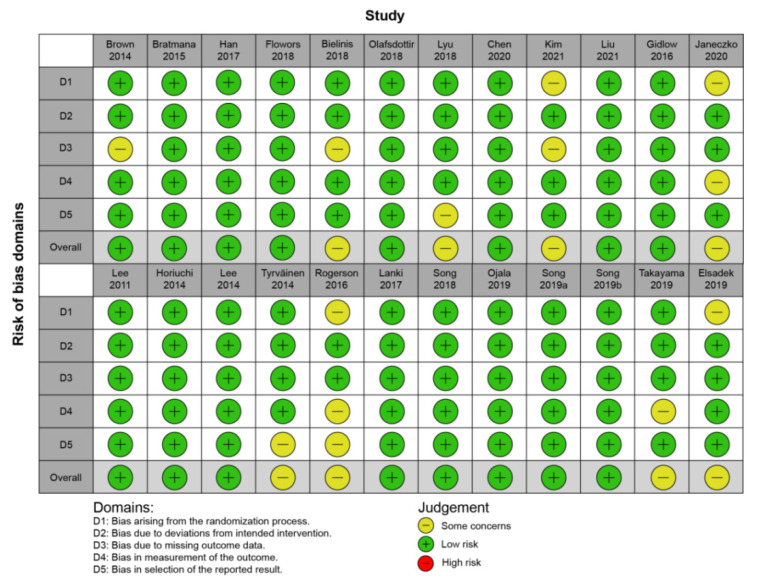
Methodological quality of included studies [26,27,28,29,30,31,32,33,34,35,36,37,38,39,40,41,42,43,44,45,46,47,48,49].

Figure 2 summarizes the results of the risk of bias assessment for the included studies. The majority of the studies had low risk and were acceptable in all five of the following categories: randomization process, deviations from the intended interventions, missing outcome data, measurement of the outcome, and selection of the reported result. Five studies had some risk. Three of them lacked means and standard deviations for the entire collection of outcome variables. The other two studies did not elaborate on the random assignment process and methodologies.

### 3.4. Outcome Variables

**Figure 3:**
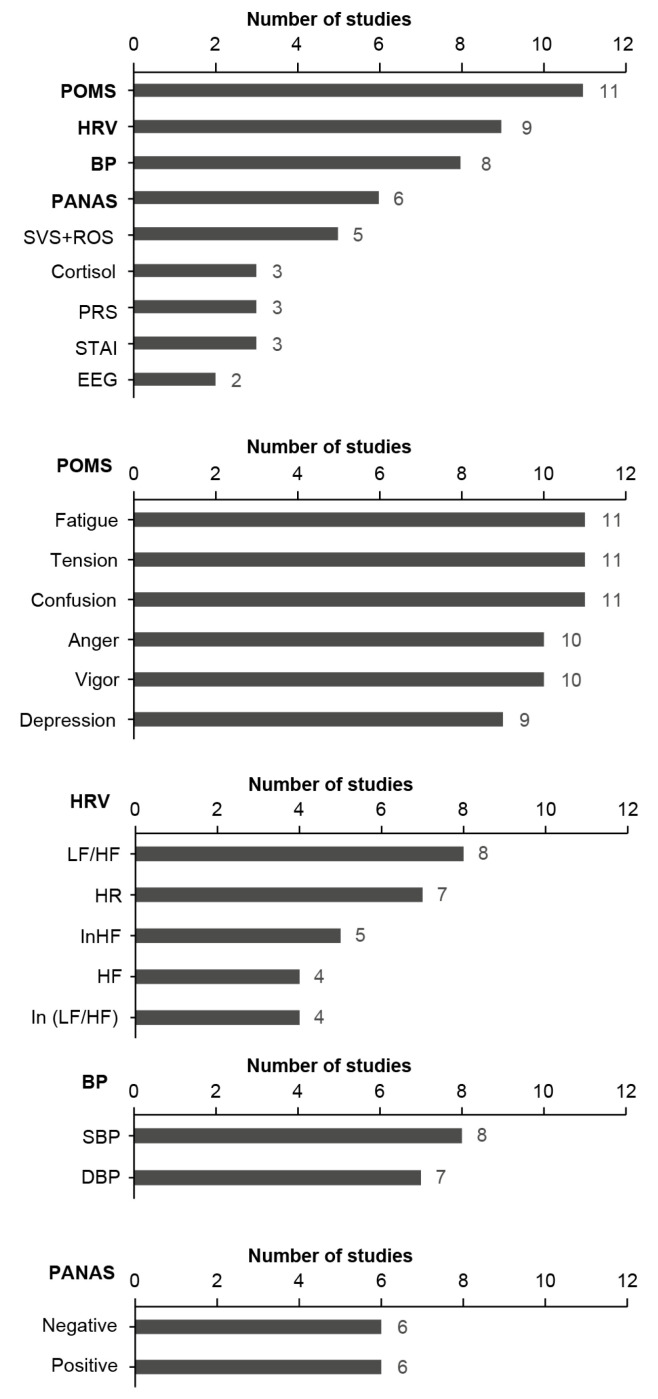
The number of studies that measured different outcome variables (total number of studies = 24). POMS: Profile of Mood States; HRV: Heart Rate Variability; BP: Blood Pressure; PANAS: Positive and Negative; SVS: Subjective Vitality Scale; ROS: Restorative Outcome Scale; PRS: Perceived Restorative Scale; STAI: State-Trait Anxiety Inventory; EEG: Electroencephalogram; HR: Heart Rate; HF: High Frequency omponent of heart rate variability; LF: Low Frequency component of heart rate variability; SBP: Systolic Blood Pressure; DBP: Diastolic Blood Pressure.

There were three primary categories of outcome variables in the 24 studies: psychological evaluations, physiological variables, and hormone levels. First, the most prevalent health restorative outcomes were measures of individual emotions, including: (1) the Profile of Mood States (POMS) of six mood states (anger, confusion, depression, fatigue, tension, and vigor); (2) the Positive and Negative Affect Schedule (PANAS); (3) the Subjective Vitality Scale (SVS); (4) the Restorative Outcome Scale (ROS); (5) the Perceived Restorative Scale (PRS); and (6) the State-Trait Anxiety Inventory (STAI) (Figure 3). Eleven of the 24 studies used POMS measures [30,34,37,38,39,40,42,43,44,46,47,48], and six used PANAS [27,30,31,37,41,48]. Many approaches, such as POMS, assess more than one emotion (e.g., depression, anger, tension).

Second, three groups of physiological variables were often considered in the 24 trials. (1) The first group included heart rate variability (HRV); heart rate (HR); the high-frequency component of heart rate variability (HF); the natural logarithm of the high-frequency component of HRV (In HF), which reflects parasympathetic activity; and the natural logarithm of the low-frequency/high-frequency ratio (In LF/HF), which reflects sympathetic activity [26,31,35,39,40,43,44,46,49]. (2) The second group included blood pressure (BP), systolic blood pressure (SBP), and diastolic blood pressure (DBP) [26,35,38,39,40,43,46,47]. (3) The third group included electroencephalogram (EEG) [33,49].

Finally, three studies examined hormone levels, namely those of cortisol [31,36,38]. Overall, the following four outcome variable categories were the most frequently used: BP (SBP, DBP), HRV, POMS scale, and PANAS scale. Hence, given that the review included studies measuring a range of different outcomes, studies measuring these four outcomes were chosen to perform a meta-analysis on particular outcomes.

### 3.5. Psychological Status Responses to Green Space Exposure

#### 3.5.1. Profile of Mood States

First, an overall effect test on the entire selected sample found that there was a high level of statistical heterogeneity in findings from trials of fatigue, tension, confusion, vigor, and depression (F: I^2^ = 82.6%, *p* = 0.000; T: I^2^ = 83.7%, *p* = 0.000; C: I^2^ = 84.5%, *p* = 0.000; V: I^2^ = 83.5%, *p* = 0.000; D: I^2^ = 83.4%, *p* = 0.000) and moderate heterogeneity in anger (I^2^ = 61.1%, *p* = 0.008) between groups with green space settings and non-green space settings.

**Figure 4:**
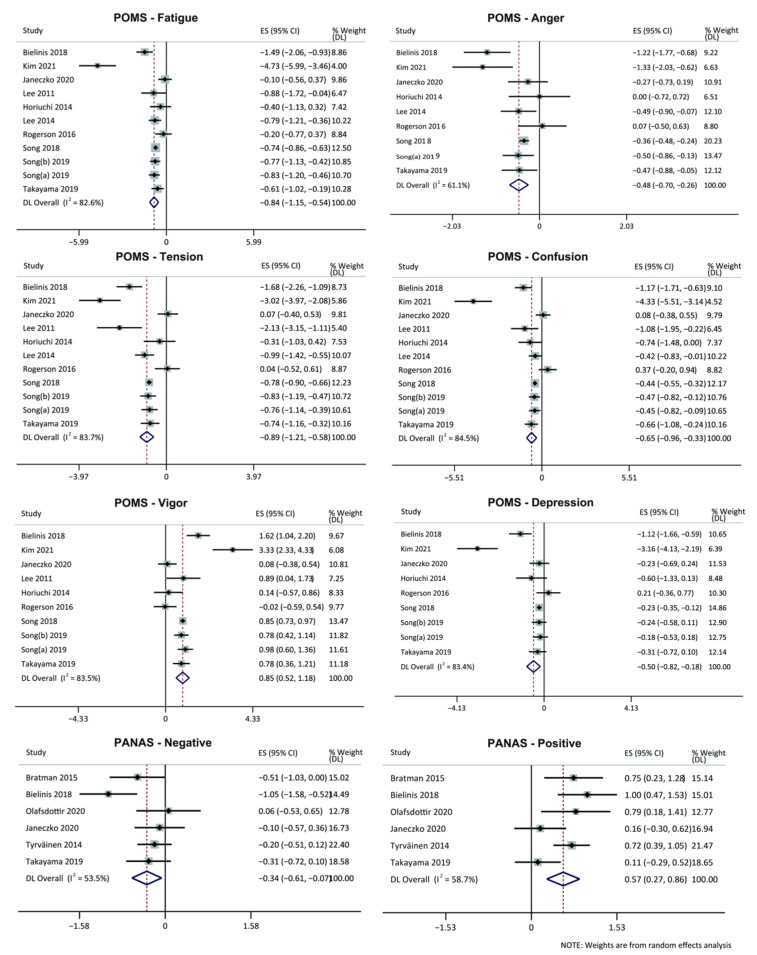
Forest plot of the meta-analysis of the relationship between green space exposure intervention and psychological indicators. Note. This forest plot depicts the meta-analysis results derived from the random effects model. In the case of POMS-Fatigue, the pooled effect size was −0.84, with a 95% confidence interval of −1.15 to −0.54. It shows that the experimental group’s Fatigue score was 0.84 lower than the control group’s and that the difference was statistically significant, *p* = 0.019 < 0.05 (Table A5). This demonstrates that a short-term green space intervention can reduce fatigue significantly. ES = effect size; I^2^ = extent of heterogeneity in effect size across studies; CI = confidence interval; POMS: Profile of Mood States; PANAS: Positive and Negative [27,30,31,34,37,38,39,40,41,42,44,46,47,48].

An overall random effects model test on the selected samples revealed that green space exposure had significant effects on alleviating fatigue, anger, tension, and confusion and enhancing vigor. Among them, fatigue and tension obtained large effect sizes of −0.84 (95% CI: −1.15 to −0.54) and −0.89, respectively, (95% CI: −1.21 to −0.58) and were statistically significant (*p* = 0.019, *p* = 0.005), indicating that green space exposure reduced fatigue and tension to a large extent; anger and confusion obtained moderate effects of −0.48 (95% CI: −0.70 to −0.26) and −0.65 (95% CI: −0.96 to −0.33), respectively, with statistical significance (*p* = 0.009, *p* = 0.048), indicating that exposure to green space significantly reduced anger and confusion. Vigor obtained a large effect size of 0.85 (95% CI: 0.52 to 1.18), with statistical significance (*p* = 0.012), indicating that green space exposure significantly increased vigor. Depression obtained a moderate effect size of −0.50 (95% CI: −0.82 to −0.18), but the results were not significant (*p* = 0.096) (Figure 4).

**Figure 5:**
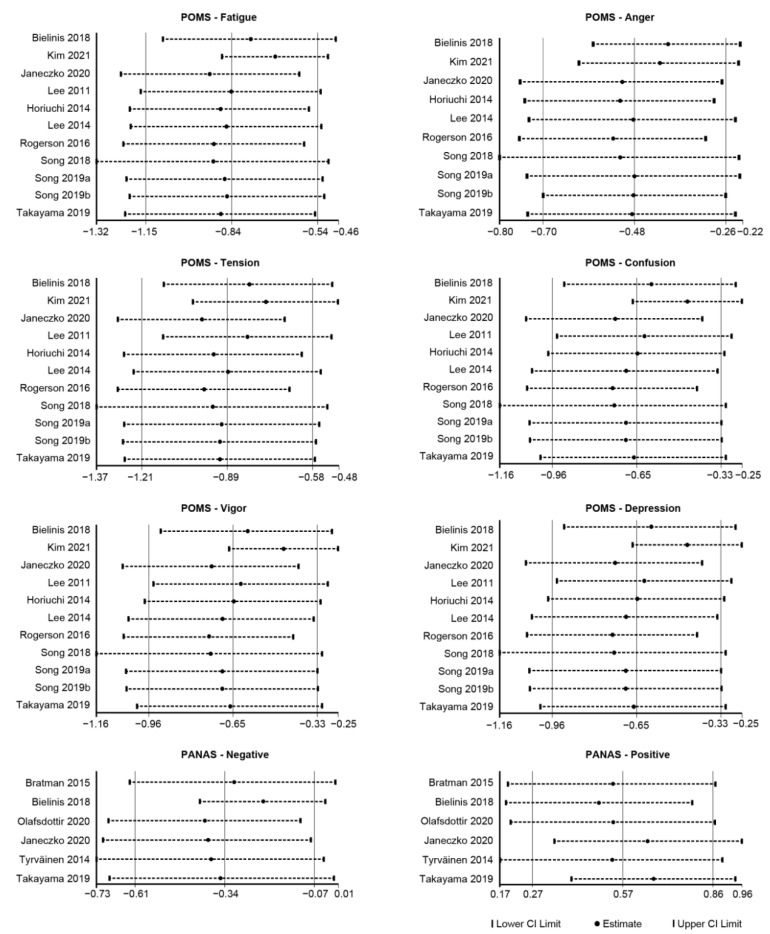
Overall sensitivity analysis of psychological outcome variables. Note. A sensitivity analysis was performed by leaving out one study item at a time and comparing the change in the pooled effect sizes before and after each item was taken out. After excluding each study, there were no significant changes in effect sizes or confidence intervals, indicating that the analysis results were relatively stable. POMS: Profile of Mood States; PANAS: Positive and Negative; CI = Confidence Interval [27,30,31,34,37,38,39,40,41,42,44,46,47,48].

Sensitivity analysis showed that these studies had little effect on the overall results (Figure 5), and they were stable and basically within the confidence interval. The Egger regression test yielded *p*-values greater than 0.05 (see Appendix A Table A6 for details), indicating that there was no publication bias in the literature of this study.

#### 3.5.2. Positive and Negative Affect Schedule

An overall effect test on the entire selected sample found that there was a moderate level of heterogeneity in negative and positive effects (I^2^ = 53.5%, *p* = 0.057, I^2^ = 58.7%, *p* = 0.034) between groups with green space settings and non-green space settings. An overall random effects model test revealed that positive effects obtained a medium effect size of 0.57 (95% CI: 0.27 to 0.86), with statistical significance (*p* = 0.013), and implying that green space exposure improved positive mood to a large extent. Meanwhile, the negative effects obtained a medium effect size of −0.34 (95% CI: −0.61 to −0.07), and the results were not significant (*p* = 0.073) (Figure 4).

Sensitivity analysis showed that these studies had little effect on the overall results (Figure 5), and the results were stable and basically within the confidence interval. The Egger regression test yielded *p* values greater than 0.05 (negative, *p* = 0.578; positive, *p* = 0.716) (see Appendix A Table A6 for details), indicating that there was no publication bias in the literature of this study.

### 3.6. Physiological Status Responses to Green Space Exposure

#### 3.6.1. Blood Pressure

**Figure 6:**
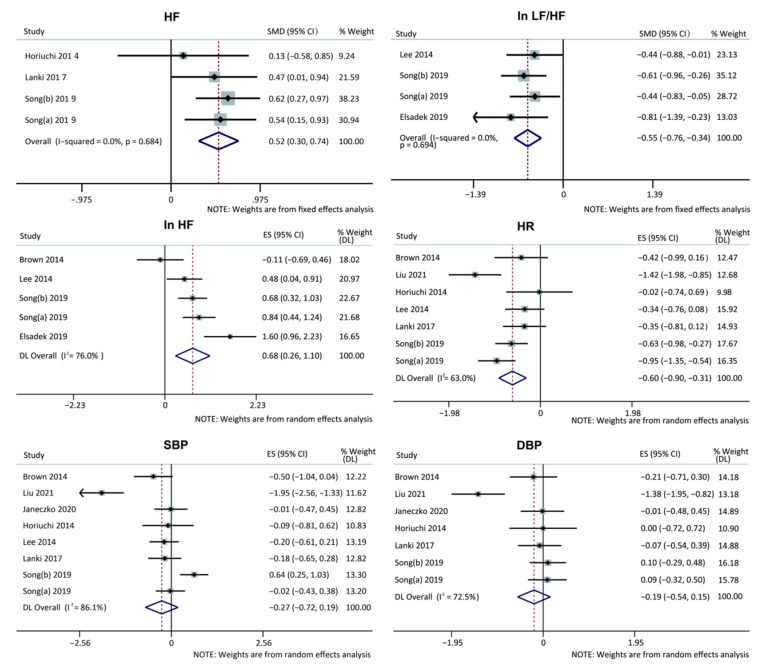
Forest plot of the meta-analysis of the relationship between green space exposure intervention and physiological indicators. Note. A heterogeneity test of the literature involved yielded I^2^ = 0 < 50% and *p* = 0.49 > 0.1 for the Q-test for the outcome variables, HF and In LF/HF. This result indicates no heterogeneity in the literature used for this study. The meta-analysis thus utilized a model with fixed effects. This analysis’s effect value is the standardized mean difference (SMD). For instance, the experimental intervention reduced In LF/HF levels with an effect size of −0.55. The other four outcome variables, In HF, HR, SBP, and DBP, were more heterogeneous in the literature (I^2^ > 50%), so a random-effects model was selected for the meta-analysis. ES = effect size; I^2^ = extent of heterogeneity in effect size across studies; CI = confidence interval; HR: Heart Rate; HF: High Frequency component of heart rate variability; LF: Low Frequency component of heart rate variability; SBP: Systolic Blood Pressure; DBP: Diastolic Blood Pressure [26,35,37,39,40,43,46,47,49].

An overall effect test on the entire selected sample found that there was a high level of heterogeneity in SBP and DBP (I^2^ = 86.1%, *p* = 0.000, I^2^ = 72.5%, *p* = 0.001,) between groups with green space settings and non-green space settings. An overall random effects model test revealed that SBP and DBP obtained a medium effect size of −0.27 (95% CI: −0.72 to 0.19) and a small effect size of −0.19 (95% CI: −0.54 to 0.15), respectively (Figure 6). Their results were not significant (*p* = 0.329, *p* = 0.348).

**Figure 7:**
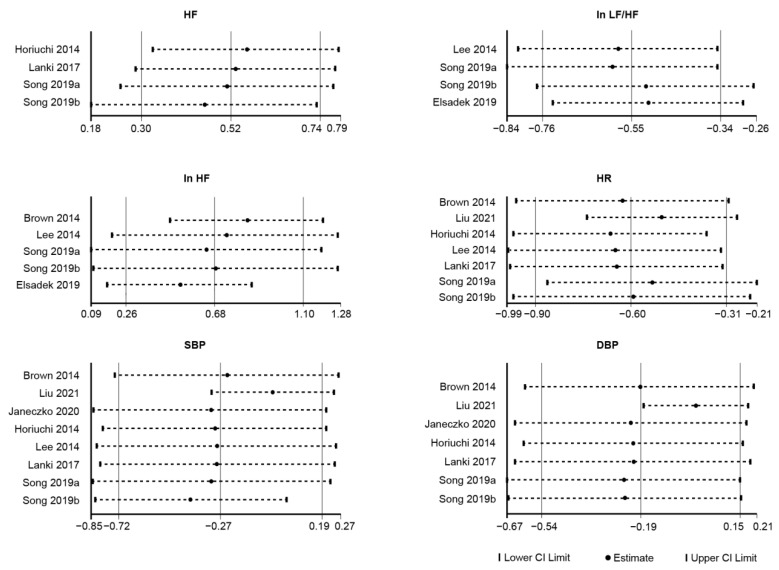
Overall sensitivity analysis of physiological outcome variables. Note. A sensitivity analysis was performed by leaving out one study item at a time and comparing the change in the pooled effect sizes before and after each item was taken out. After excluding each study, there were no significant changes in effect sizes or confidence intervals, indicating that the analysis results were relatively stable. HR: Heart Rate; HF: High Frequency component of heart rate variability; LF: Low Frequency component of heart rate variability; SBP: Systolic Blood Pressure; DBP: Diastolic Blood Pressure; CI = confidence interval [26,35,37,39,40,43,46,47,49].

Sensitivity analysis showed that these studies had little effect on the overall results (Figure 7), and the results were stable and basically within the confidence interval. The Egger regression test yielded *p* values greater than 0.05 (SBP, *p* = 0.103; DBP, *p* = 0.275) (see Appendix A Table A6 for details), indicating that there was no publication bias in the literature of this study.

#### 3.6.2. Heart Rate Variability

First, an overall effect test on the entire selected sample found that there was a low level of statistical heterogeneity in the findings of HF and In LF/HF (I^2^ = 0.0%, *p* = 0.684; I^2^ = 0.0%, *p* = 0.694), moderate heterogeneity in HR (I^2^ = 63%, *p* = 0.013), and high heterogeneity in In HF (I^2^ = 76%, *p* = 0.002) between groups with green space settings and non-green space settings.

An overall fixed effects model test on the selected samples revealed that HF obtained a medium effect of 0.52 (95% CI: 0.30 to 0.74), with statistical significance (*p* = 0.018), indicating that exposure in green space significantly increased HF. In LF/HF obtained a medium effect of −0.55 (95% CI: −0.76 to −0.34), with statistical significance (*p* = 0.014), indicating that exposure in green space significantly reduced In LF/HF. An overall random effects model test on the selected samples revealed that In HF obtained a medium effect size of 0.68 (95% CI: 0.26 to 1.1), and the results were not significant (*p* = 0.06). HR obtained a medium effect of −0.60 (95% CI: −0.90 to −0.31), with statistical significance (*p* = 0.011), indicating that exposure to green space significantly reduced HR (Figure 6).

Sensitivity analysis showed that these studies had little effect on the overall results (Figure 7), and the results were stable and basically within the confidence interval. The Egger regression test yielded *p* values greater than 0.05, except for HF (see Appendix A Table A6 for details).

## 4. Discussion

### 4.1. Findings concerning the Research Questions

This review aimed to synthesize evidence from randomized controlled trials regarding the physiological and psychological effects of direct exposure to green space. Our systematic review and meta-analysis included 24 studies. Overall, there is evidence that direct contact with activities in greenspaces can improve public mental health. First, positive health effects were demonstrated by a meta-analysis of self-reported mood results from several studies. Exposure to green space was associated with lower negative feelings, such as exhaustion, anger, tension, and confusion, than exposure to non-green space environments. In addition, green space exposure was associated with higher positive feelings, such as notably improved vigor. Second, according to the available evidence, exposure to green space can considerably lower physiological indicators such as In LF/HF and HR levels. This persistent difference was not supported by a meta-analysis of other physiological indicator variables, such as blood pressure. Each analysis was based on more than four studies. 

Out of these 24 studies, the POMS scale, the PANAS scale, the HRV index, and blood pressure were often used as outcome variables. The following nine outcome indicators were statistically significantly changed before and after the intervention, POMS-Fatigue −0.84 (−1.15, −0.54) (*p* < 0.05), POMS-Tension −0.89 (−1.21, −0.58) (*p* < 0.05), POMS-Vigor 0.85 (0.52, 1.18) (*p* < 0.05), POMS-Anger −0.48 (−0.70, −0.26) (*p* < 0.05), POMS-Confusion −0.65 (−0.96, −0.33) (*p* < 0.05), PANAS-Positive 0.57 (0.27,0.86) (*p* < 0.05), HF 0.52 (0.30,0.74) (*p* < 0.05), In LF/HF −0.55 (−0.76, −0.34) (*p* < 0.05) and HR −0.60 (−0.90, −0.31) (*p* < 0.05). There were three with large effect sizes (POMS-Fatigue, POMS-Tension, and POMS-Vigor), and the remaining six were all at moderate effect size levels. These outcome indicators and effects can be used as a reference for a future RCT study on the influence of green space intervention on mental health.

Our work differs from existing systematic reviews and meta-analysis studies in the same category in three major respects. First, we focused on natural environment exposure rather than virtual environment stimulation. To broaden the scope of green space research, compared to an earlier review study [18], we not only focused on forests but also covered a variety of other green space types, such as parks, urban woodlands, and campus green space. The extended breadth of the study helps to better investigate the health benefits of accessible green space. Second, we focused on RCT studies, which further decreased the heterogeneity of the study design in comparison to earlier reviews [50,51,52,53,54], resulting in more accurate pooled effect values. Finally, unlike previous studies, we focused on mental health in the general population rather than physical exercise and specific disorders, to generalize our findings to a large population.

To our knowledge, this is the first systematic review and meta-analysis is to evaluate the benefits of real green space exposure on mental health restoration in RCTs. A 2018 meta-analysis examined the relationship between exposure to green space and human health, including systolic and diastolic blood pressure and heart rate [3]. The results indicated that exposure to outdoor green space significantly altered heart rate values. The meta-values of HR were estimated to be stronger in our analysis than in the study by Twohig-Bennett and Jones. The varying numbers and features of the included studies may contribute to this disparity. In comparison, Twohig-Bennett and Jones’ study design was more complex. They considered 103 observational studies and 40 intervention trials; in addition, there were 35 cohort studies and 69 cross-sectional studies among the observational studies. Our study only included RCTs to reduce meta-analysis heterogeneity.

### 4.2. Limitations and Future Research

Because the available RCT experimental designs mostly focus on urban versus natural contexts, our meta-analysis is insufficient for comparing different types of green space. Additionally, green space indicators were not addressed in our study, and further RCTs in various green space are required to acquire data for meta-analysis of the outcomes. Moreover, in terms of activity type and environmental interaction mode, the current RCT experimental investigations are limited and thus insufficient to provide subgroup analyses comparing activity type and spatial type. Finally, the findings may result in some bias because existing RCTs on greenspace exposure have paid insufficient attention to interventions for older and adolescent groups, different ethnic groups, different regions, and greenspace exposure doses.

Therefore, we provide several recommendations for future research:

(1) More RCT-based experimental investigations are needed to reveal the potential mechanisms and causal relationships between green space characteristics and mental health.
(2) The types of green spaces could be further refined to compare their effect sizes and minimal effects in the future.
(3) We recommend that researchers replicate these small-scale experiments in diverse geographic regions and subgroups.
(4) In order to correct current biases, future research should pay more attention to underage and elderly populations, multiethnic samples, long-term exposure interventions, and doses of greenspace interventions.

## 5. Conclusions

This study’s quantitative synthesis showed that green space exposure could induce physical and psychological relaxation. Our meta-analysis showed that exposure to green space significantly impacted fatigue, anger, tension, confusion, vitality, positive affect measured using the PANAS scale, HR, and In LF/HF levels. In addition, we summarized the outcome variable indicators with high effect size, such as POMS-fatigue, and indicators with medium effect size, such as In LF/HF. We suggest that future RCT studies select appropriate outcome variables based on these results to study further the impact of green space intervention dose on restoration. Our research results can provide support and reference for the formulation of green space intervention measures and design strategies in the future. In the studies included in this review, almost all indicators except HF have no publication bias. However, future research should include different geographic regions, other age groups, races, and long-term exposure interventions to correct current biases.

## Figures and Tables

**Table 1:** Characteristics of Studies Included in Meta-analysis.

No.	Studies	Participants				GSE ^1^ Intervention			Types of GS
		E ^2^ (n)	C ^3^ (n)	Age	Male/Female	Experimental Group	Control Group	Frequency and Duration	
1	(Brown et al., 2014) [26]	32	62	42 ± 10.6	74/20	GS ^4^ walking	non-GS walking	20 min, 2 times/week for 8 weeks	country lanes with trees
2	(Bratman et al., 2015) [27]	30	30	22.9	27/33	GS walking	street walking	50 min	Urban park
3	(Han, 2017) [28]	58	58	20.85 ± 1.14	52/64	GS walking	non-GS walking	15 min	Campus green space
4	(Flowers et al., 2018) [29]	30	30	19.9 ± 4.26	19/41	GS cycling	indoor cycling	15 min	grassland
5	(Bielinis et al., 2018) [30]	31	31	21.45 ± 0.18	36/26	GS viewing	non-GS viewing	15 min	forest
6	(Olafsdottir et al., 2020) [31]	20	23	24.39 ± 2.61	21/46	GS walking	non-GS walking	40 min	woodland
7	(Lyu et al., 2019) [32]	90	30	22.1 ± 0.4	60/60	GS viewing + walking	non-GS viewing + walking	15 min viewing + 15 min walking	forest
8	(Chen et al., 2020) [33]	16	16	20.6 ± 1.6	16/16	GS viewing	non-GS viewing	20 min	wooded garden
9	(Kim et al., 2021) [34]	19	19	22.1 ± 1.6	24/14	GS activities	normal daily life	once a week for 8 sessions	forest
10	(Liu et al., 2021) [35]	30	30	23.9 ± 1.86	14/16	GS sitting + walking	non-GS sitting + walking	30 min sitting + 30 min walking	forest
11	(Gidlow et al., 2016) [36]	38	38	40.9 ± 17.6	23/15	GS walking	non-GS walking	30 min	Urban park
12	(Janeczko et al., 2020) [37]	E1 = 17, E2 = 13	C1 = 23, C2 = 22	19–24	not clear	GS walking	non-GS walking	30 min	forest
13	(Lee et al., 2011) [38]	12	12	21.2 ± 0.9	12/0	GS viewing	non-GS viewing	15 min	forest
14	(Horiuchi et al., 2014) [39]	15	15	36 ± 8	11/4	GS viewing	non-GS viewing	15 min	forest
15	(Lee et al., 2014) [40]	48	48	21.1 ± 1.2	48/0	GS walking	non-GS walking	12–15 min	forest
16	(Tyrvainen et al., 2014) [41]	77	77	47.64 ± 8.68	6/71	GS viewing + walking	urban viewing + walking	15 min viewing + 30 min walking	forest
17	(Rogerson et al., 2016) [42]	24	24	35.1 ± 20.1	5/19	GS cycling	indoor cycling	15 min	grassland
18	(Lanki et al., 2017) [43]	36	36	46 ± 8.7	0/36	GS viewing + walking	urban viewing + walking	15 min viewing + 30 min walking	forest
19	(Song et al., 2018) [44]	585	585	21.7 ± 1.6	585/0	GS walking	non-GS walking	15 min	forest
20	(Ojala et al., 2019) [45]	83	83	48.31 ± 8.58	0/83	GS viewing + walking	urban viewing + walking	15 min viewing + 30 min walking	forest
21	(Song et al., 2019a) [46]	60	60	21.0 ± 1.3	0/60	GS walking	non-GS walking	15 min	forest
22	(Song et al., 2019b) [47]	65	65	21.0 ± 1.3	0/65	GS viewing	non-GS viewing	15 min	forest
23	(Takayama et al., 2019) [48]	46	46	21.12	46/0	GS walking + viewing	non-GS walking + viewing	15 min walking + 15 min viewing	forest
24	(Elsadek et al., 2019) [49]	25	25	23 ± 1.5	0/25	GS viewing	non-GS viewing	5 min	green façade

^1^ GSE, green space exposure; ^2^ E, experimental group; ^3^ C, control group; ^4^ GS, green space.

## Data Availability

Not applicable.

## Appendix A

**Table A1:** Search content used for systematic search on evidence for the restorative benefits of exposure to green spaces on PubMed database (updated on 6 December 2021).

No.	Query	Results
11	((((“Parks, Recreational”[Mesh]) OR (((((((((((((((((((((((((((((((((((((((((((((((open spaces[Title/Abstract]) OR (green spaces[Title/Abstract])) OR (outdoor[Title/Abstract])) OR (parks[Title/Abstract])) OR (Recreational Park[Title/Abstract])) OR (Recreational Parks[Title/Abstract])) OR (National Parks[Title/Abstract])) OR (National Park[Title/Abstract])) OR (Park, National[Title/Abstract])) OR (Parks, National[Title/Abstract])) OR (Community Parks[Title/Abstract])) OR (Community Park[Title/Abstract])) OR (Park, Community[Title/Abstract])) OR (Parks, Community[Title/Abstract])) OR (Green Space[Title/Abstract])) OR (Space, Green[Title/Abstract])) OR (Urban Parks[Title/Abstract])) OR (Park, Urban[Title/Abstract])) OR (Parks, Urban[Title/Abstract])) OR (Urban Park[Title/Abstract])) OR (Forests[Title/Abstract])) OR (Forested Areas[Title/Abstract])) OR (Area, Forested[Title/Abstract])) OR (Areas, Forested[Title/Abstract])) OR (Forested Area[Title/Abstract])) OR (Woodland[Title/Abstract])) OR (Woodlands[Title/Abstract])) OR (Forestlands[Title/Abstract])) OR (Forestland[Title/Abstract])) OR (blue space[Title/Abstract])) OR (blue spaces[Title/Abstract])) OR (wetlands[Title/Abstract])) OR (wetland[Title/Abstract])) OR (pond[Title/Abstract])) OR (ponds[Title/Abstract])) OR (fountain[Title/Abstract])) OR (Lake[Title/Abstract])) OR (Lakes[Title/Abstract])) OR (Soda Lakes[Title/Abstract])) OR (Lakes, Soda[Title/Abstract])) OR (Rivers[Title/Abstract])) OR (River[Title/Abstract])) OR (water[Title/Abstract])) OR (coast[Title/Abstract])) OR (canal[Title/Abstract])) OR (Streams[Title/Abstract])) OR (Stream[Title/Abstract]))) AND ((“Exercise”[Mesh]) OR ((((((((((((((((((((((((((((((exposure[Title/Abstract]) OR (access[Title/Abstract])) OR (walk[Title/Abstract])) OR (walking[Title/Abstract])) OR (cycle[Title/Abstract])) OR (exercises[Title/Abstract])) OR (Physical Activity[Title/Abstract])) OR (Activities, Physical[Title/Abstract])) OR (Activity, Physical[Title/Abstract])) OR (Physical Activities[Title/Abstract])) OR (Exercise, Physical[Title/Abstract])) OR (Exercises, Physical[Title/Abstract])) OR (Physical Exercise[Title/Abstract])) OR (Physical Exercises[Title/Abstract])) OR (Acute Exercise[Title/Abstract])) OR (Acute Exercises[Title/Abstract])) OR (Exercise, Acute[Title/Abstract])) OR (Exercises, Acute[Title/Abstract])) OR (Exercise, Isometric[Title/Abstract])) OR (Exercises, Isometric[Title/Abstract])) OR (Isometric Exercises[Title/Abstract])) OR (Isometric Exercise[Title/Abstract])) OR (Exercise, Aerobic[Title/Abstract])) OR (Aerobic Exercise[Title/Abstract])) OR (Aerobic Exercises[Title/Abstract])) OR (Exercises, Aerobic[Title/Abstract])) OR (Exercise Training[Title/Abstract])) OR (Exercise Trainings[Title/Abstract])) OR (Training, Exercise[Title/Abstract])) OR (Trainings, Exercise[Title/Abstract])))) AND ((“Mental Health”[Mesh]) OR ((((((((((((Health, Mental[Title/Abstract]) OR (Mental Hygiene[Title/Abstract])) OR (Hygiene, Mental[Title/Abstract])) OR (restoration[Title/Abstract])) OR (physiological effects[Title/Abstract])) OR (psychological effects[Title/Abstract])) OR (mood[Title/Abstract])) OR (stress[Title/Abstract])) OR (wellbeing[Title/Abstract])) OR (blood pressure[Title/Abstract])) OR (heart rate[Title/Abstract])) OR (depression[Title/Abstract])))) AND (randomized controlled trial[Publication Type] OR randomized[Title/Abstract] OR placebo[Title/Abstract])	839
10	randomized controlled trial[Publication Type] OR randomized[Title/Abstract] OR placebo[Title/Abstract]	923,605
9	(“Mental Health”[Mesh]) OR ((((((((((((Health, Mental[Title/Abstract]) OR (Mental Hygiene[Title/Abstract])) OR (Hygiene, Mental[Title/Abstract])) OR (restoration[Title/Abstract])) OR (physiological effects[Title/Abstract])) OR (psychological effects[Title/Abstract])) OR (mood[Title/Abstract])) OR (stress[Title/Abstract])) OR (wellbeing[Title/Abstract])) OR (blood pressure[Title/Abstract])) OR (heart rate[Title/Abstract])) OR (depression[Title/Abstract]))	1,834,651
8	(((((((((((Health, Mental[Title/Abstract]) OR (Mental Hygiene[Title/Abstract])) OR (Hygiene, Mental[Title/Abstract])) OR (restoration[Title/Abstract])) OR (physiological effects[Title/Abstract])) OR (psychological effects[Title/Abstract])) OR (mood[Title/Abstract])) OR (stress[Title/Abstract])) OR (wellbeing[Title/Abstract])) OR (blood pressure[Title/Abstract])) OR (heart rate[Title/Abstract])) OR (depression[Title/Abstract])	1,806,935
7	“Mental Health”[Mesh]	49,226
6	(“Exercise”[Mesh]) OR ((((((((((((((((((((((((((((((exposure[Title/Abstract]) OR (access[Title/Abstract])) OR (walk[Title/Abstract])) OR (walking[Title/Abstract])) OR (cycle[Title/Abstract])) OR (exercises[Title/Abstract])) OR (Physical Activity[Title/Abstract])) OR (Activities, Physical[Title/Abstract])) OR (Activity, Physical[Title/Abstract])) OR (Physical Activities[Title/Abstract])) OR (Exercise, Physical[Title/Abstract])) OR (Exercises, Physical[Title/Abstract])) OR (Physical Exercise[Title/Abstract])) OR (Physical Exercises[Title/Abstract])) OR (Acute Exercise[Title/Abstract])) OR (Acute Exercises[Title/Abstract])) OR (Exercise, Acute[Title/Abstract])) OR (Exercises, Acute[Title/Abstract])) OR (Exercise, Isometric[Title/Abstract])) OR (Exercises, Isometric[Title/Abstract])) OR (Isometric Exercises[Title/Abstract])) OR (Isometric Exercise[Title/Abstract])) OR (Exercise, Aerobic[Title/Abstract])) OR (Aerobic Exercise[Title/Abstract])) OR (Aerobic Exercises[Title/Abstract])) OR (Exercises, Aerobic[Title/Abstract])) OR (Exercise Training[Title/Abstract])) OR (Exercise Trainings[Title/Abstract])) OR (Training, Exercise[Title/Abstract])) OR (Trainings, Exercise[Title/Abstract]))	2,153,311
5	(“Parks, Recreational”[Mesh]) OR (((((((((((((((((((((((((((((((((((((((((((((((open spaces[Title/Abstract]) OR (green spaces[Title/Abstract])) OR (outdoor[Title/Abstract])) OR (parks[Title/Abstract])) OR (Recreational Park[Title/Abstract])) OR (Recreational Parks[Title/Abstract])) OR (National Parks[Title/Abstract])) OR (National Park[Title/Abstract])) OR (Park, National[Title/Abstract])) OR (Parks, National[Title/Abstract])) OR (Community Parks[Title/Abstract])) OR (Community Park[Title/Abstract])) OR (Park, Community[Title/Abstract])) OR (Parks, Community[Title/Abstract])) OR (Green Space[Title/Abstract])) OR (Space, Green[Title/Abstract])) OR (Urban Parks[Title/Abstract])) OR (Park, Urban[Title/Abstract])) OR (Parks, Urban[Title/Abstract])) OR (Urban Park[Title/Abstract])) OR (Forests[Title/Abstract])) OR (Forested Areas[Title/Abstract])) OR (Area, Forested[Title/Abstract])) OR (Areas, Forested[Title/Abstract])) OR (Forested Area[Title/Abstract])) OR (Woodland[Title/Abstract])) OR (Woodlands[Title/Abstract])) OR (Forestlands[Title/Abstract])) OR (Forestland[Title/Abstract])) OR (blue space[Title/Abstract])) OR (blue spaces[Title/Abstract])) OR (wetlands[Title/Abstract])) OR (wetland[Title/Abstract])) OR (pond[Title/Abstract])) OR (ponds[Title/Abstract])) OR (fountain[Title/Abstract])) OR (Lake[Title/Abstract])) OR (Lakes[Title/Abstract])) OR (Soda Lakes[Title/Abstract])) OR (Lakes, Soda[Title/Abstract])) OR (Rivers[Title/Abstract])) OR (River[Title/Abstract])) OR (water[Title/Abstract])) OR (coast[Title/Abstract])) OR (canal[Title/Abstract])) OR (Streams[Title/Abstract])) OR (Stream[Title/Abstract]))	1,132,218
4	(((((((((((((((((((((((((((((exposure[Title/Abstract]) OR (access[Title/Abstract])) OR (walk[Title/Abstract])) OR (walking[Title/Abstract])) OR (cycle[Title/Abstract])) OR (exercises[Title/Abstract])) OR (Physical Activity[Title/Abstract])) OR (Activities, Physical[Title/Abstract])) OR (Activity, Physical[Title/Abstract])) OR (Physical Activities[Title/Abstract])) OR (Exercise, Physical[Title/Abstract])) OR (Exercises, Physical[Title/Abstract])) OR (Physical Exercise[Title/Abstract])) OR (Physical Exercises[Title/Abstract])) OR (Acute Exercise[Title/Abstract])) OR (Acute Exercises[Title/Abstract])) OR (Exercise, Acute[Title/Abstract])) OR (Exercises, Acute[Title/Abstract])) OR (Exercise, Isometric[Title/Abstract])) OR (Exercises, Isometric[Title/Abstract])) OR (Isometric Exercises[Title/Abstract])) OR (Isometric Exercise[Title/Abstract])) OR (Exercise, Aerobic[Title/Abstract])) OR (Aerobic Exercise[Title/Abstract])) OR (Aerobic Exercises[Title/Abstract])) OR (Exercises, Aerobic[Title/Abstract])) OR (Exercise Training[Title/Abstract])) OR (Exercise Trainings[Title/Abstract])) OR (Training, Exercise[Title/Abstract])) OR (Trainings, Exercise[Title/Abstract])	2,064,571
3	“Exercise”[Mesh]	222,284
2	((((((((((((((((((((((((((((((((((((((((((((((open spaces[Title/Abstract]) OR (green spaces[Title/Abstract])) OR (outdoor[Title/Abstract])) OR (parks[Title/Abstract])) OR (Recreational Park[Title/Abstract])) OR (Recreational Parks[Title/Abstract])) OR (National Parks[Title/Abstract])) OR (National Park[Title/Abstract])) OR (Park, National[Title/Abstract])) OR (Parks, National[Title/Abstract])) OR (Community Parks[Title/Abstract])) OR (Community Park[Title/Abstract])) OR (Park, Community[Title/Abstract])) OR (Parks, Community[Title/Abstract])) OR (Green Space[Title/Abstract])) OR (Space, Green[Title/Abstract])) OR (Urban Parks[Title/Abstract])) OR (Park, Urban[Title/Abstract])) OR (Parks, Urban[Title/Abstract])) OR (Urban Park[Title/Abstract])) OR (Forests[Title/Abstract])) OR (Forested Areas[Title/Abstract])) OR (Area, Forested[Title/Abstract])) OR (Areas, Forested[Title/Abstract])) OR (Forested Area[Title/Abstract])) OR (Woodland[Title/Abstract])) OR (Woodlands[Title/Abstract])) OR (Forestlands[Title/Abstract])) OR (Forestland[Title/Abstract])) OR (blue space[Title/Abstract])) OR (blue spaces[Title/Abstract])) OR (wetlands[Title/Abstract])) OR (wetland[Title/Abstract])) OR (pond[Title/Abstract])) OR (ponds[Title/Abstract])) OR (fountain[Title/Abstract])) OR (Lake[Title/Abstract])) OR (Lakes[Title/Abstract])) OR (Soda Lakes[Title/Abstract])) OR (Lakes, Soda[Title/Abstract])) OR (Rivers[Title/Abstract])) OR (River[Title/Abstract])) OR (water[Title/Abstract])) OR (coast[Title/Abstract])) OR (canal[Title/Abstract])) OR (Streams[Title/Abstract])) OR (Stream[Title/Abstract])	1,131,931
1	“Parks, Recreational”[Mesh]	1749

**Table A2:** Search content used for systematic search on evidence for the restorative benefits of exposure to green spaces on Web of Science database (updated on 6 December 2021).

No.	Query	Results
5	(((#1) AND #2) AND #3) AND #4	2785
4	TS = (randomized controlled trial or randomized or placebo)	1,301,005
3	TS = (exposure or access or walk or walking or cycle or exercise or Exercises or Physical Activity or Activities, Physical or Activity, Physical or Physical Activities or Exercise, Physical or Exercises, Physical or Physical Exercise or Physical Exercises or Acute Exercise or Acute Exercises or Exercise, Acute or Exercises, Acute or Exercise, Isometric or Exercises, Isometric or Isometric Exercises or Isometric Exercise or Exercise, Aerobic or Aerobic Exercise or Aerobic Exercises or Exercises, Aerobic or Exercise Training or Exercise Trainings or Training, Exercise or Trainings, Exercise)	8,763,397
2	TS = (green spaces or open spaces or outdoor or parks or Park, Recreational or Recreational Park or Recreational Parks or National Parks or National Park or Park, National or Parks, National or Community Parks or Community Park or Park, Community or Parks, Community or Green Space or Space, Green or Urban Parks or Park, Urban or Parks, Urban or Urban Park or Forests or Forested Areas or Area, Forested or Areas, Forested or Forested Area or Woodland or Woodlands or Forestlands or Forestland or blue space or wetland or pond or fountain or Lake or Soda Lakes or Lakes, Soda or River or water or coast or canal or Streams or Stream)	12,881,797
1	TS = (mental health or Health, Mental or Mental Hygiene or Hygiene, Mental or restoration or physiological effects or psychological effects or mood or stress or wellbeing or blood pressure or heart rate or depression)	7,169,338

**Table A3:** Search content used for systematic search on evidence for the restorative benefits of exposure to green spaces on Embase database (updated on 6 December 2021).

No.	Query	Results
#11	#3 AND #6 AND #9 AND #10	749
#10	‘randomized controlled trial’:ab,ti OR ‘randomized’:ab,ti OR ‘placebo’:ab,ti	1,010,050
#9	#7 OR #8	2,409,252
#8	‘access’:ab,ti OR ‘walk’:ab,ti OR ‘walking’:ab,ti OR ‘cycle’:ab,ti OR ‘exercise’:ab,ti OR ‘exercises’:ab,ti OR ‘physical activity’:ab,ti OR ‘activities, physical’:ab,ti OR ‘activity, physical’:ab,ti OR ‘physical activities’:ab,ti OR ‘exercise, physical’:ab,ti OR ‘exercises, physical’:ab,ti OR ‘physical exercise’:ab,ti OR ‘physical exercises’:ab,ti OR ‘acute exercise’:ab,ti OR ‘acute exercises’:ab,ti OR ‘exercise, acute’:ab,ti OR ‘exercises, acute’:ab,ti OR ‘exercise, isometric’:ab,ti OR ‘exercises, isometric’:ab,ti OR ‘isometric exercises’:ab,ti OR ‘isometric exercise’:ab,ti OR ‘exercise, aerobic’:ab,ti OR ‘aerobic exercise’:ab,ti OR ‘aerobic exercises’:ab,ti OR ‘exercises, aerobic’:ab,ti OR ‘exercise training’:ab,ti OR ‘exercise trainings’:ab,ti OR ‘training, exercise’:ab,ti OR ‘trainings, exercise’:ab,ti	1,765,422
#7	‘exposure’/exp	677,420
#6	#4 OR #5	1,313,802
#5	‘green spaces’:ab,ti OR ‘open spaces’:ab,ti OR ‘outdoor’:ab,ti OR ‘parks’:ab,ti OR ‘park, recreational’:ab,ti OR ‘recreational park’:ab,ti OR ‘recreational parks’:ab,ti OR ‘national parks’:ab,ti OR ‘national park’:ab,ti OR ‘park, national’:ab,ti OR ‘parks, national’:ab,ti OR ‘community parks’:ab,ti OR ‘community park’:ab,ti OR ‘park, community’:ab,ti OR ‘parks, community’:ab,ti OR ‘green space’:ab,ti OR ‘space, green’:ab,ti OR ‘urban parks’:ab,ti OR ‘park, urban’:ab,ti OR ‘parks, urban’:ab,ti OR ‘urban park’:ab,ti OR ‘forests’:ab,ti OR ‘forested areas’:ab,ti OR ‘area, forested’:ab,ti OR ‘areas, forested’:ab,ti OR ‘forested area’:ab,ti OR ‘woodland’:ab,ti OR ‘woodlands’:ab,ti OR ‘forestlands’:ab,ti OR ‘forestland’:ab,ti OR ‘blue space’:ab,ti OR ‘wetland’:ab,ti OR ‘pond’:ab,ti OR ‘fountain’:ab,ti OR ‘lake’:ab,ti OR ‘soda lakes’:ab,ti OR ‘lakes, soda’:ab,ti OR ‘river’:ab,ti OR ‘water’:ab,ti OR ‘coast’:ab,ti OR ‘canal’:ab,ti OR ‘streams’:ab,ti OR ‘stream’:ab,ti	131,3796
#4	‘green space’/exp	99
#3	#1 OR #2	24,38,836
#2	‘health, mental’:ab,ti OR ‘mental hygiene’:ab,ti OR ‘hygiene, mental’:ab,ti OR ‘restoration’:ab,ti OR ‘physiological effects’:ab,ti OR ‘psychological effects’:ab,ti OR ‘mood’:ab,ti OR ‘stress’:ab,ti OR ‘wellbeing’:ab,ti OR ‘blood pressure’:ab,ti OR ‘heart rate’:ab,ti OR ‘depression’:ab,ti	2,318,173
#1	‘mental health’/exp	190,151

**Table A4:** Search content used for systematic search on evidence for the restorative benefits of exposure to green spaces on Scopus database (updated on 6 December 2021).

Search Within	Search Documents
Article title, Abstract, Keywords	“mental health” OR “Health, Mental” OR “Mental Hygiene” OR “Hygiene, Mental” OR “restoration “ OR “physiological effects” OR “psychological effects” OR “mood” OR “stress” OR “wellbeing” OR “blood pressure” OR “heart rate” OR “depression”
AND	
Search within	Search documents
Article title, Abstract, Keywords	“green space” OR “green spaces” OR “open spaces” OR “outdoor” OR “parks” OR “Park, Recreational” OR “Recreational Park” OR “Recreational Parks” OR “National Parks” OR “National Park” OR “Park, National” OR “Parks, National” OR “Community Parks” OR “Community Park” OR “Park, Community” OR “Parks, Community” OR “Green Space” OR “Space, Green” OR “Urban Parks” OR “Park, Urban” OR “Parks, Urban” OR “Urban Park” OR “Forests” OR “Forested Areas” OR “Area, Forested” OR “Areas, Forested” OR “Forested Area” OR “Woodland” OR “Woodlands” OR “Forestlands” OR “Forestland” OR “blue space” OR “wetland” OR “pond” OR “fountain” OR “Lake” OR “Soda Lakes” OR “Lakes, Soda” OR “River” OR “water” OR “coast” OR “canal” OR “Streams” OR “Stream”
AND	
Search within	Search documents
Article title, Abstract, Keywords	“exposure” OR “access” OR “walk” OR “walking” OR “cycle” OR “exercise” OR “Exercises” OR “Physical Activity” OR “Activities, Physical” OR “Activity, Physical” OR “Physical Activities” OR “Exercise, Physical” OR “Exercises, Physical” OR “Physical Exercise” OR “Physical Exercises” OR “Acute Exercise” OR “Acute Exercises” OR “Exercise, Acute” OR “Exercises, Acute” OR “Exercise, Isometric” OR “Exercises, Isometric” OR “Isometric Exercises” OR “Isometric Exercise” OR “Exercise, Aerobic” OR “Aerobic Exercise” OR “Aerobic Exercises” OR “Exercises, Aerobic” OR “Exercise Training” OR “Exercise Trainings” OR “Training, Exercise” OR “Trainings, Exercise”
AND	
Search within	Search documents
Article title, Abstract, Keywords	“randomized controlled trial” OR “randomized” OR “placebo”

**Table A5:** Standardized effect size significance evaluation.

POMS							
A	_ES|	exp(b)	Std. Err.	t	*P* > |t|	[95% Conf. Interval]	
	_cons|	0.6151379	0.0866342	−3.45	**0.009**	0.4445558	0.8511747
C	_ES|	exp(b)	Std. Err.	t	*P* > |t|	[95% Conf. Interval]	
	_cons|	0.4634556	0.1585505	−2.25	**0.048**	0.2162539	0.9932358
D	_ES|	exp(b)	Std. Err.	t	*P* > |t|	[95% Conf. Interval]	
	_cons|	0.5559821	0.1730284	−1.89	0.096	0.2712606	1.139554
F	_ES|	exp(b)	Std. Err.	t	*P* > |t|	[95% Conf. Interval]	
	_cons|	0.3818658	0.1316885	−2.79	**0.019**	0.1770945	0.8234107
T	_ES|	exp(b)	Std. Err.	t	*P* > |t|	[95% Conf. Interval]	
	_cons|	0.386793	0.102785	−3.57	**0.005**	0.2139606	0.6992355
V	_ES|	exp(b)	Std. Err.	t	*P* > |t|	[95% Conf. Interval]	
	_cons|	2.447043	0.7020197	3.12	**0.012**	1.278773	4.682626
PANAS							
NEG	_ES|	exp(b)	Std. Err.	t	*P* > |t|	[95% Conf. Interval]	
	_cons|	0.7117126	0.1066662	−2.27	0.073	0.4841594	1.046215
POS	_ES|	exp(b)	Std. Err.	t	*P* > |t|	[95% Conf. Interval]	
	_cons|	1.765879	0.2663032	3.77	**0.013**	1.198406	2.602064
BP							
SBP	_ES|	exp(b)	Std. Err.	t	*P* > |t|	[95% Conf. Interval]	
	_cons|	0.7627319	0.1970277	−1.05	0.329	0.4140888	1.404916
DBP	_ES|	exp(b)	Std. Err.	t	*P* > |t|	[95% Conf. Interval]	
	_cons|	0.8211954	0.1590445	−1.02	0.348	0.5112491	1.319048
HRV							
InHF	_ES|	exp(b)	Std. Err.	t	*P* > |t|	[95% Conf. Interval]	
	_cons|	1.98176	0.5210963	2.60	0.060	0.9549746	4.112541
In LF/HF	_ES|	exp(b)	Std. Err.	t	*P* > |t|	[95% Conf. Interval]	
	_cons|	0.5778081	0.0614678	−5.16	**0.014**	0.4118626	0.8106155
HF	_ES|	exp(b)	Std. Err.	t	*P* > |t|	[95% Conf. Interval]	
	_cons|	1.681798	0.1868437	4.68	**0.018**	1.180929	2.395101
HR	_ES|	exp(b)	Std. Err.	t	*P* > |t|	[95% Conf. Interval]	
	_cons|	0.5466781	0.0902664	−3.66	**0.011**	0.3649758	0.8188404

**Table A6:** Results of Egger’s test.

POMS							
A	Number of studies = 9		Root MSE = 1.623				
	Std_Eff |	Coef.	Std. Err.	t	*P* > |t|	[95% Conf. Interval]	
	sloPe |	−0.3119461	0.1295039	−2.41	0.047	−0.6181742	−0.0057179
	bias |	−0.7939601	0.8843085	−0.90	0.399	−2.885017	1.297097
	Test of H0: no small−study effects		*P* = 0.399				
C	Number of studies = 11		Root MSE = 2.493				
	Std_Eff |	Coef.	Std. Err.	t	*P* > |t|	[95% Conf. Interval]	
	sloPe |	−0.2910019	0.1903744	−1.53	0.161	−0.7216586	0.1396549
	bias |	−1.430026	1.215727	−1.18	0.270	−4.18019	1.320139
	Test of H0: no small−study effects		*P* = 0.270				
D	Number of studies = 9		Root MSE = 2.289				
	Std_Eff |	Coef.	Std. Err.	t	*P* > |t|	[95% Conf. Interval]	
	sloPe |	−0.0718342	0.1792568	−0.40	0.701	−0.4957092	0.3520407
	bias |	−1.836489	1.238808	−1.48	0.182	−4.765806	1.092827
	Test of H0: no small−study effects		*P* = 0.182				
F	Number of studies = 11		Root MSE = 2.456				
	Std_Eff |	Coef.	Std. Err.	t	*P* > |t|	[95% Conf. Interval]	
	sloPe |	−0.631019	0.1922104	−3.28	0.009	−1.065829	−0.1962088
	bias |	−0.8942754	1.20419	−0.74	0.477	−3.618344	1.829793
	Test of H0: no small−study effects		*P* = 0.477				
T	Number of studies = 11		Root MSE = 2.551				
	Std_Eff |	Coef.	Std. Err.	t	*P* > |t|	[95% Conf. Interval]	
	sloPe |	−0.6800191	0.2008553	−3.39	0.008	−1.134385	−0.2256528
	bias |	−0.8096559	1.253264	−0.65	0.534	−3.644736	2.025424
	Test of H0: no small−study effects		*P* = 0.534				
V	Number of studies = 10		Root MSE = 2.608				
	Std_Eff |	Coef.	Std. Err.	t	*P* > |t|	[95% Conf. Interval]	
	sloPe |	0.8015194	0.2082304	3.85	0.005	0.3213392	1.2817
	bias |	0.1736208	1.324815	0.13	0.899	−2.881409	3.22865
	Test of H0: no small−study effects		*P* = 0.899				
PANAS							
NEG	Number of studies = 6		Root MSE = 1.569				
	Std_Eff |	Coef.	Std. Err.	t	*P* > |t|	[95% Conf. Interval]	
	sloPe |	0.0820798	0.6749266	0.12	0.909	−1.791817	1.955976
	bias |	−1.811976	2.993059	−0.61	0.578	−10.12204	6.498087
	Test of H0: no small−study effects		*P* = 0.578				
POS	Number of studies = 6		Root MSE = 1.707				
	Std_Eff |	Coef.	Std. Err.	t	*P* > |t|	[95% Conf. Interval]	
	sloPe |	0.2669537	0.7555394	0.35	0.742	−1.83076	2.364667
	bias |	1.290405	3.305122	0.39	0.716	−7.886084	10.46689
	Test of H0: no small−study effects		*P* = 0.716				
BP							
SBP	Number of studies = 8		Root MSE = 2.278				
	Std_Eff |	Coef.	Std. Err.	t	*P* > |t|	[95% Conf. Interval]	
	sloPe |	1.842106	1.051731	1.75	0.130	−0.731388	4.415599
	bias |	−8.366707	4.354697	−1.92	0.103	−19.02227	2.288853
	Test of H0: no small−study effects		*P* = 0.103				
DBP	Number of studies = 7		Root MSE = 1.831				
	Std_Eff |	Coef.	Std. Err.	t	*P* > |t|	[95% Conf. Interval]	
	sloPe |	0.9924329	0.9391591	1.06	0.339	−1.421753	3.406618
	bias |	−4.757269	3.881868	−1.23	0.275	−14.73593	5.221389
	Test of H0: no small−study effects		*P* = 0.275				
HRV							
InHF	Number of studies = 5		Root MSE = 2.345				
	Std_Eff |	Coef.	Std. Err.	t	*P* > |t|	[95% Conf. Interval]	
	sloPe |	0.4804744	1.141271	0.42	0.702	−3.151558	4.112507
	bias |	0.8432633	5.00189	0.17	0.877	−15.07498	16.76151
	Test of H0: no small−study effects		*P* = 0.877				
In LF/HF	Number of studies = 4		Root MSE = 0.7369				
	Std_Eff |	Coef.	Std. Err.	t	*P* > |t|	[95% Conf. Interval]	
	sloPe |	−0.1806944	0.4576711	−0.39	0.731	−2.149894	1.788506
	bias |	−1.754716	2.151093	−0.82	0.500	−11.01012	7.50069
	Test of H0: no small−study effects		*P* = 0.500				
HF	Number of studies = 4		Root MSE = 0.0932				
	Std_Eff |	Coef.	Std. Err.	t	*P* > |t|	[95% Conf. Interval]	
	sloPe |	1.073631	0.0437214	24.56	0.002	0.8855133	1.26175
	bias |	−2.5652	0.1967704	−13.04	0.006	−3.411835	−1.718565
	Test of H0: no small−study effects		*P* = 0.006				
HR	Number of studies = 7		Root MSE = 1.782				
	Std_Eff |	Coef.	Std. Err.	t	*P* > |t|	[95% Conf. Interval]	
	sloPe |	−0.8518194	0.7435951	−1.15	0.304	−2.763291	1.059653
	bias |	1.014648	3.133803	0.32	0.759	−7.04105	9.070345
	Test of H0: no small−study effects		*P* = 0.759				
